# Of Mice, Cattle, and Humans: The Immunology and Treatment of River Blindness

**DOI:** 10.1371/journal.pntd.0000217

**Published:** 2008-04-30

**Authors:** Judith E. Allen, Ohene Adjei, Odile Bain, Achim Hoerauf, Wolfgang H. Hoffmann, Benjamin L. Makepeace, Hartwig Schulz-Key, Vincent N. Tanya, Alexander J. Trees, Samuel Wanji, David W. Taylor

**Affiliations:** 1 Institute for Immunology and Infection Research, University of Edinburgh, Edinburgh, United Kingdom; 2 Kwame Nkrumah University of Science and Technology, Kumasi, Ghana; 3 Museum National d'Histoire Naturelle, Paris, France; 4 Universitätsklinikum Bonn, Bonn, Germany; 5 Eberhard Karls Universität, Tübingen, Germany; 6 Liverpool School of Tropical Medicine and Faculty of Veterinary Science, University of Liverpool, Liverpool, United Kingdom; 7 Institut de Recherche Agricole pour le Développement, Ngaoundéré, Cameroon; 8 Research Foundation in Tropical Diseases and Environment, Buea, Cameroon; 9 Centre for Infectious Diseases, Royal (Dick) School for Veterinary Studies, University of Edinburgh, Edinburgh, United Kingdom; New York Blood Center, United States of America

## Abstract

River blindness is a seriously debilitating disease caused by the filarial parasite *Onchocerca volvulus*, which infects millions in Africa as well as in South and Central America. Research has been hampered by a lack of good animal models, as the parasite can only develop fully in humans and some primates. This review highlights the development of two animal model systems that have allowed significant advances in recent years and hold promise for the future. Experimental findings with *Litomosoides sigmodontis* in mice and *Onchocerca ochengi* in cattle are placed in the context of how these models can advance our ability to control the human disease.

## Introduction

**Figure 1:**
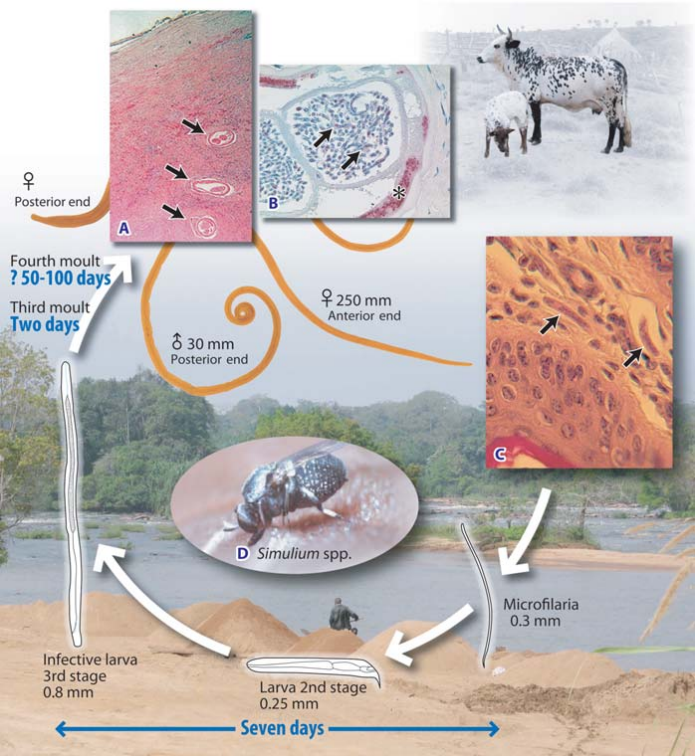
Life cycle of *Onchocerca volvulus* and *Onchocerca ochengi*. Adult female worms initiate the formation of nodules in the skin (onchocercomas) (see Figures 2 and 3) in which their highly coiled bodies can reach a length of approximately 25 cm, while the males are a little over 1/10th that length. Transverse sections of adult female worms in the onchocercoma are shown in (A). Following mating, embryos develop inside the female, which gives birth to motile L1 larvae that are known as microfilaria (MF). A transverse section of an adult female with MF in utero is shown in (B); *Wolbachia* in lateral hypodermal chords (*) of the adult female and uterine microfilaria (arrows) are stained red. MF migrate into the dermis (shown in [C]), where they are available for transmission to the simuliid blackfly vector (shown in [D]). Within the fly, MF develop further as L1 larvae and molt into second-stage larvae, which molt again to become the infective L3 larvae (7 days). The L3 enter the skin through the wound caused by the feeding fly. The blackfly requires fast moving water to breed and thus infection occurs adjacent to rivers. Adult female worms live for several years and individuals (people or cattle) can remain microfilaraemic for their entire lives if repeatedly exposed to infection. (Photo credits: M. Boussinesq, S. Spetch, J. Allen, O Bain, S. Wanji, S. Uni)

**Figure 2:**
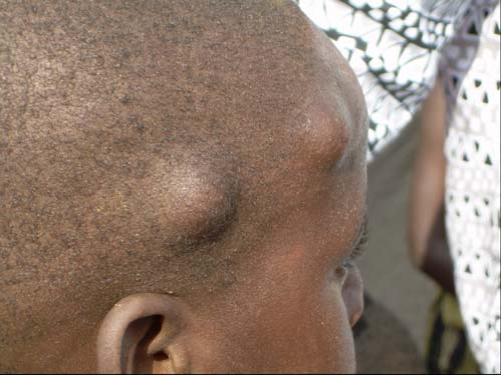
Subcutaneous Nodules on a Child in Ghana. Photo credit: P. Soboslay.

Infection with *Onchocerca volvulus*, a filarial nematode, can lead to debilitating skin disease and blindness (river blindness). Adult worms live in subcutaneous nodules; however, the pathology of onchocerciasis is primarily associated with death of microfilariae larvae in the skin and eyes (Figures 1 and 2). It is estimated that 37 million people are infected with *O. volvulus* [1], over 99% of whom live in West and Central Africa, although there are significant foci in South and Central America. Early attempts at control of onchocerciasis relied on treatment of water courses with insecticides to kill the larvae (larviciding) of the blackfly (*Simulium* spp.) vectors. Using this approach for over 25 years, the WHO/UNDP Onchocerciasis Control Programme (OCP) reduced the burden of disease in savannah regions of West Africa [2],[3]. In 1987, ivermectin (Mectizan, Merck & Co.) was introduced for mass treatment of onchocerciasis either alone or in combination with larviciding. The OCP closed in December 2002, and control of onchocerciasis now relies on community-based treatment with ivermectin implemented through the African Programme for Onchocerciasis Control (APOC) [4]. The Onchocerciasis Elimination Programme for the Americas similarly distributes Mectizan twice a year in its target countries of Brazil, Colombia, Ecuador, Guatemala, Mexico, and Venezuela [5].

Ivermectin is very effective at killing microfilariae and has proved successful in reducing morbidity within the community and the risk of severe skin or ocular disease for the individual. However, its macrofilaricidal activity (i.e., efficacy against adult parasites) is, at best, slow and partial, necessitating the use of repeated drug administration for several years [6]–[8]. Furthermore, early hopes that mass treatment with ivermectin would eradicate the disease by breaking transmission have not been realised [2] because of inadequate treatment coverage, migration, and recrudescence of infections in areas where treatment has been suspended. In addition, there is mounting evidence that resistance to ivermectin is emerging [9]–[13]. Such circumstances require development of complementary measures to sustain even the current levels of control, let alone eliminate the disease. What are needed is a safe and effective macrofilaricide and a vaccine.

A major obstacle facing onchocerciasis research and, particularly that concerned with vaccine development, has been the absence of good animal models. Use of mice was limited because they are unable to support cyclical development of filariae species. All rodents are strictly non-permissive to *O. volvulus*, which can develop only in primates, and thus studies of protective immunity in mice involve implantation of infective stage larvae (L3) into subcutaneous chambers [14]. Mice are somewhat more permissive to *Brugia* species (causative agents of lymphatic filariasis) but still do not allow natural tissue migration or development of circulating microfilariae. Patent infections with circulating microfilariae can be established in the Mongolian gerbil (*Meriones unguiculatus*) with *Brugia* species and *Acanthocheilonema viteae*; however, the absence of reagents places serious restrictions on immunological investigation. Nonetheless, despite limitations, these models have made significant contributions to our knowledge of filarial infections (reviewed in [14]–[16]) and provided a basis of more recent investigations using two new models.

The first is *Litomosoides sigmodontis* (Table 1), a natural parasite of the cotton rat (*Sigmodon hispidus*) that in the early 1990s was found to undergo complete development in BALB/c mice and produce patent infections with circulating microfilariae within 55–60 days post-infection [17]. Development of *L. sigmodontis* in other inbred strains is restricted. For example, in C57BL/6 mice, filariae are progressively killed and never produce a patent infection. It is now possible to utilise the full power of murine immunology to study the interaction of filarial parasites with their hosts at all stages of the parasite's development from migration of infective larvae to the production of microfilariae. The ability of *L. sigmodontis* to achieve patency allows a comparison to human studies not possible in other murine models. The data thus far show a striking similarity to human studies, particularly in the context of regulation (discussed below); thus, through experimental manipulation, this model can provide mechanistic explanations of susceptibility and resistance not possible in any other system.

**Table 1:** General Features of the Biology of *O. volvulus*, *O. ochengi*, and *L. sigmodontis*

Filariae	Vector	Time to Patency	Adult	Mf	Disadvantages	Advantages
*O. volvulus*	Blackfly, *Simulium* spp.	250–375 days	Subcutaneous nodules	Skin	Experimentation not possible	The target organism
*O. ochengi*	Blackfly, *Simulium* spp.	From 250 days	Intradermal nodules	Skin	Outbred animals, no pathology	–Very closely related to *O. volvulus*
						–Experimentation under natural challenge
						–Infection quantifiable
*L. sigmodontis*	Tropical rat mite, *Ornithonyssus bacoti*	∼50 days	Thoracic cavity	Blood	Not skin dwelling, no pathology	–All stages of the life cycle accessible for experimentation
						–Power of murine immunology
						–Protective immunity evoked by vaccination

**Figure 3:**
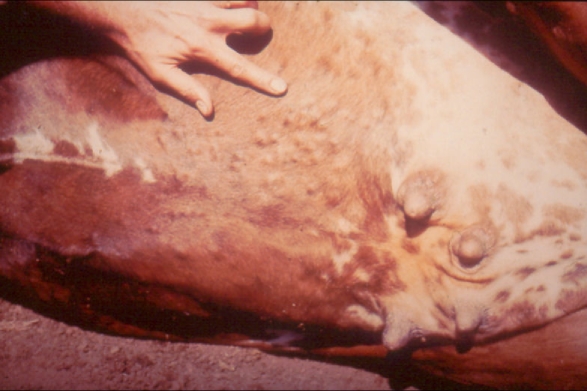
Intradermal Nodules Containing Adult *Onchocerca ochengi* on Ventral Hide of a Naturally Infected Cow (*Bos indicus*) in Cameroon. Photo credit: A. J. Trees.

The second model is *Onchocerca ochengi* in cattle (Table 1; Figure 3). This is the closest known relative of the human parasite and is also transmitted by the blackfly, *Simulium damnosum sensu lato*. *O. ochengi* is confined to Africa and combines many important features of the human infection [18]. Most importantly, *O. ochengi* forms nodules that closely resemble those of *O. volvulus* and which can be enumerated non-invasively or removed for analysis during immunological or chemotherapeutic studies. Furthermore, putative immune animals exist naturally in endemic areas and exhibit demonstrable resistance to infection [19]. The *O. ochengi* model thus provides the unique opportunity to undertake controlled experiments, both laboratory-based and under natural challenge in the field, that are not possible in humans.

The main drawback to both of these model systems is that they do not allow the investigation of disease pathology relevant to human onchocerciasis. This work will continue to rely on either human field studies or experimental exposure of mice to *Onchocerca* antigens in a model of ocular disease [20].

## Mechanisms of Parasite Killing

In the *L. sigmodontis* model, innate responses at the inoculation site are associated with destruction of a majority of L3s in the subcutaneous tissue within 2 days post-infection. However, about one-third of L3 larvae avoid this attack by entering lymphatic vessels [21],[22], a strategy characteristic of many human filariae [23],[24]. The number of larvae that survive this early stage varies depending on sex and strain of the host [25], but is unaffected by the size of the initial inoculum [26]. From Day 4 post-inoculation, surviving L3 begin to appear in the pleural cavity of *L. sigmodontis*–infected mice. Differences in the pattern of development of the parasites in resistant C57BL/6 and susceptible BALB/c mice appear early and get progressively more apparent [25]. By 30 days post-infection, about one-third of the population in C57BL/6 mice are still at the L4 stage; this contrasts with <15% in susceptible BALB/c mice [27]. Furthermore, worms recovered from the C57BL/6 mice are smaller than those from BALB/c mice. Analysis of cytokine production at this time shows mixed T helper cell type 1 (Th1)-Th2 response in the C57BL/6 mice reminiscent of that observed in putative immune human patients [28]. In BALB/c mice, the cytokine response is more biased towards Th2 (see Box 1).

**Box 1. Th1 & Th2 Immunity**

Helminth parasites are typically associated with the induction of CD4+ T helper 2 (Th2) cells, while microbial pathogens induce Th1 responses. Filarial parasites constitute a unique stimulus to the immune system, as they are worms that (in most cases) contain endosymbiotic bacteria (*Wolbachia*). The Th1 response functions to activate macrophages to be more efficient at microbial destruction and is essential to survive infection with many intracellular pathogens such as *Mycobacteria* and *Salmonella*. The Th2 response is involved in expelling worms from the intestines as well as encapsulating and destroying multicellular parasites. The Th2 response also plays a key role in wound healing and allergic reactions. Macrophages are mediators of both Th1 and Th2 immunity but exhibit different functions. Mast cells and eosinophils are dependent on Th2 cytokines for expansion and recruitment. Th1 and Th2 responses are also associated with differing antibody isotype profiles, with Th1 cytokines promoting cytophilic antibodies while Th2 responses promote antibodies involved in allergic-type responses such as IgE. In addition to T helper cells, T regulatory subsets exist that function primarily to prevent host damage caused by overactive effector responses. These are associated with the production of TGF-β and/or IL-10. Neutrophils, not pictured here, are phagocytic cells of the innate immune system that may become activated prior to Th1/Th2 polarisation, but are also strongly associated with a fourth CD4+ T helper subset: Th17 cells. Th17 cells are strongly pro-inflammatory and have roles in mediating autoimmune disease as well as protection against extracellular bacteria and may exacerbate pathology during helminth infection (for a review of T helper subsets in helminth infection, see Díaz and Allen [87]).

**Figure:**
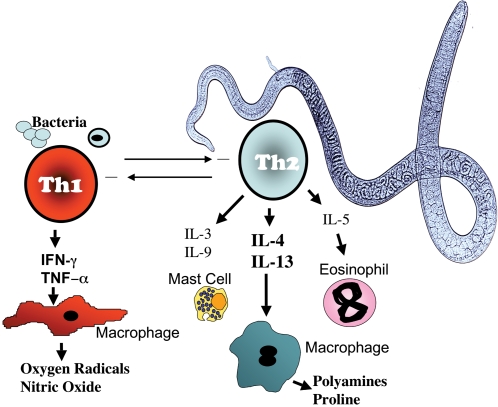

The ability of filarial parasites to induce Th2-type immune responses is well documented, but whether this bias is detrimental or beneficial for the parasite is not always clear. However, infection of IL-4–deficient C57BL/6 mice leads to full parasite development and patency, indicating that a Th2 response is the key determinant of resistance in these non-permissive mice [29]. Consistent with a role for type 2 immunity in parasite killing, partially resistant 129/SvJ mice with a genetic deficiency in either major basic protein or eosinophil peroxidase were found to harbour several times more adult worms than their wild-type littermates [30]. Further, BALB/c mice deficient in IL-4, IL-5, or IL-4Rα (unable to respond to IL-4 or IL-13) present with levels of microfilariae 100 times higher than wild-type controls [31],[32]. This evidence that type 2 cytokines can control microfilarial levels is consistent with studies on *Brugia* species [33].

Although the data began to build a convincing argument for Th2 control of filarial infections, the picture that emerged proved more complex. The BALB/c IL-4Rα–deficient mice presented a paradox, for although the mice had enormously increased numbers of circulating microfilaria relative to wild-type mice, death of the adult parasites was accelerated. Examination of the effector cells at the site of infection demonstrated that the mice had converted to a Th1 phenotype suggesting that a pro-inflammatory type 1 response was capable of killing the adult parasite (J. Allen and M. G. Nair, unpublished data). Consistent with this, more adult worms are recovered from mice genetically deficient in the type 1 cytokine, IFN-γ [34],[35] Indeed, IFN-γ and IL5 appear to act synergistically to destroy adult parasites [34],[36]. Thus, although Th2 responses seem capable of mediating destruction of the larval stages, both Th1 and Th2 may be needed to contain the more resilient adult stage.

Induction of a Th1 response may be a consequence of the presence of the endosymbiont bacterium *Wolbachia* found in most human-pathogenic filariae [37]. Filarial-infected humans, cattle, and mice demonstrate significant immune responses to the major surface protein (WSP) of the bacteria [38],[39] (B. Makepeace and A. Trees, unpublished data). Further, WSP as well as the bacteria in total have been shown to stimulate a typical TLR-dependent inflammatory response with induction of IL-6, TNF, etc. by macrophages [40]–[42] and exhibit potent chemotactic activity for neutrophils [41]. Mice with a natural mutation of TLR4 (C3H/HeJ) show a higher degree of susceptibility to *L. sigmodontis* infection [43]. This is consistent with protection studies in the *O. volvulus* mouse chamber model that identified a TLR4-dependent larval killing mechanism, albeit with no evidence for *Wolbachia* involvement [44]. The costs and benefits of symbiosis with *Wolbachia* for filariae in terms of manipulation of host immune responses have yet to be investigated in depth. However, elimination of *Wolbachia* from *O. ochengi* leads to a profound reduction in local neutrophilia in the nodule and a marked infiltration of eosinophils, which degranulate on the cuticle of adult worms prior to parasite death [45]. This is compatible with a potential role for *Wolbachia* in modulating the anti-nematode response.

## Regulation

Although a clearer picture of how mammalian hosts can kill filarial nematodes is emerging, in a successful infection these mechanisms fail. Human studies have long since demonstrated that filarial parasites induce a state of hypo-responsiveness in the host that is associated with the presence of circulating microfilaria [46]. Both the *L. sigmodontis* and *O. ochengi* models can mimic this, with Th1 and Th2 cytokines down-regulated coincident with the onset of patency [47],[48]. Intrinsic defects in T cell responses in human filarial infection are linked with expression of the T cell–inhibiting receptor, CTLA-4 [49], and neutralisation of CTLA-4 in mice results in enhanced *L. sigmodontis* killing [50]. In addition to this intrinsic T cell hypo-responsiveness, T cell responses in humans can be dampened by suppressive antigen-presenting cells [51]. Both mechanisms are operative in the *L. sigmodontis* model where macrophages that block proliferation of T cells are present at the site of infection prior to patency but become apparent in the draining lymph nodes only following patency [52]. Studies in susceptible BALB/c mice have now directly demonstrated that *L. sigmodontis* survival is dependent on the induction of a regulatory T cell population that induces hypo-responsiveness [48]. This corroborates the data from human field studies demonstrating that T regulatory (Treg) cells can be isolated from onchocerciasis patients [53], and generalised onchocerciasis is associated with antigen-specific Treg cells that can be found in nodules [54]. These studies demonstrate the utility of the *L. sigmodontis* model to reveal details of protective and regulatory mechanisms that can help explain observations made in human infections.

The importance of immune regulation in parasite survival is also illustrated by the study of mechanisms that determine microfilarial survival. Different inbred strains of mice differ widely in their capacity to eliminate circulating microfilariae, and these genetically determined differences can be attributed to a single gene locus [55]. However, irrespective of host genetic background, microfilarial density is regulated by the adult female [56]. An immune regulatory environment with interleukin 10 (IL-10) as a key player is induced by the female parasite to facilitate the survival and persistence of her offspring [56]. In the absence of IL-4, normally resistant C57BL/6 mice develop patent infection, but the additional knock-out of IL-10 reverts mice back to a resistant phenotype [57]. This suggests that IL-10 is inhibiting an anti-worm effector response that is redundant when a full Th2 response is in place. In this scenario, wild-type C57BL/6 are non-permissive because Th2 immunity prevents worm development and patency. In the absence of IL-4, patency occurs because Th2-dependent mechanisms are absent but IL-10 is present, suppressing alternative, potentially innate, effector responses. In the absence of both cytokines, IL-10 restraint of innate mechanisms is lifted and once again worms are targeted by the immune response. Consistent with a role for IL-10 in suppressing effector responses, transgenic overexpression of IL-10 in macrophages in genetically resistant FVB mice leads to patency [36]. Design of effective vaccines must take into account that destruction of each parasite stage may require activation of distinct effector pathways and that the parasites themselves induce powerful regulatory networks to modulate these pathways.

## Vaccine-Mediated Immunity

The ability of irradiated L3 to generate protection in naïve animals challenged experimentally with normal larvae has been demonstrated in numerous models of filariasis [14],[58], including both the *L. sigmodontis* [21],[58],[59] and *O. ochengi* models [19]. The protective efficacy of irradiated L3 has been successfully translated into a field trial using *O. ochengi* in cattle in which significantly lower worm burdens were observed in vaccinated animals compared to controls after almost 2 years of continuous exposure to intense natural challenge from infected *Simulium* [19]. This success contrasts with the failure of cattle to develop immunity after drug-abbreviated infections. When naïve, infection-free calves were exposed to sustained and intensive levels of natural challenge, monthly or 3-monthly prophylaxis with macrocyclic lactones completely prevented the development of adult worms. However, when chemotherapy ended but exposure continued, these animals were found to be more susceptible to infection than previously unexposed controls [60] both in terms of adult numbers and microfilarial levels. Similarly, following successful macrofilaricidal treatment of pre-existing patent infections with melarsomine [19] or oxytetracycline [61], cattle were fully susceptible to re-infection. These data suggest that parasite death is an insufficient stimulus for the induction of protective immunity and highlight the importance of defining the mechanisms by which irradiated L3 induce protection.

The *L. sigmodontis* system allows the careful study of vaccine-mediated protection, including larval migration as early as 6 hours post-infection or challenge, as well as the impact on subsequent development and ability to develop patent infection. Immune protection generated by irradiated *L. sigmodontis* larvae leads to rapid destruction of the challenge larvae in the subcutaneous tissue [21],[62] and protection is long-lived [63]. Studies with gene-deficient mice showed that vaccination success depends on IL-5 and antibody [22],[59], and this is consistent with observations made using the *O. volvulus* mouse chamber model [64]. Evidence suggests that the pattern of migration of irradiated L3 does not differ from that of untreated L3 in the first 2 weeks of infection [62]. Further, in normal infections a high proportion of incoming larvae die and yet this does not afford protection to re-infection. These findings argue against protection as a consequence of premature parasite death or aberrant migration. L3 larvae of filarial parasites are known to induce regulatory pathways [65], and irradiated L3 may be failing to produce molecules that initiate downregulatory pathways in the host. Conversely (but not mutually exclusive) irradiated larvae may be failing to shut down the expression of early genes and thus potentially overexpress immunogenic molecules. Powerful genomic and proteomic tools are now available to address this question and to this end, extensive expressed sequence tag (EST) analysis of *L. sigmodontis* stage-specific genes is well underway [66],[67], which will help to identify both targets of immunity as well as potential immune regulators.

Because disease is associated with the microfilarial stage in onchocerciasis and because this stage is the key to transmission, an anti-microfilarial vaccine also needs to be considered. Indeed, vaccination with microfilariae of *O. lienalis* in a bovine system was shown some years ago to enhance the clearance of microfilariae subsequently transplanted into the same animal [68]. Moreover, in natural infections of cattle with *O. ochengi*, skin microfilarial density falls with age in spite of increasing numbers of fecund female worms, which suggests a level of stage-specific microfilarial immunity may develop [69]. Similar experiments using mice as a surrogate host of *O. volvulus* demonstrated that microfilariae of the human parasite are vulnerable to immune killing and that these responses can be evoked by related species; in this case, *O. lienalis* [70]. However, what is also clear is that female worms can and do modulate these protective responses [56], and that for any anti-microfilarial vaccine to be effective it must target these parasite regulatory molecules as well as the microfilarial antigens. While there are many reports identifying potential regulatory molecules [71], the search is by no means over. Identification of both parasite-derived immunomodulators and the relevant microfilarial-specific targets is now being facilitated by the filarial genome project [72] and *L. sigmodontis* EST analysis [66]. The *L. sigmodontis* and *O. ochengi* models offer powerful complementary systems to test these candidates in carefully controlled laboratory settings and field settings under natural challenge.

## Alternative Treatments Targeting *Wolbachia*

Control of onchocerciasis in Africa relies on mass distribution of microfilaricidal ivermectin. Given the impossibility of onchocerciasis eradication with ivermectin alone [2] and rising concerns about resistance to this drug [9]–[13], there is a more pressing need to identify complementary therapy using existing drugs. The development of a new drug, apart from the enormous costs, would take 15 years or more to be completed.

Attention has focused on *Wolbachia*, the bacterial endosymbionts found in most filarial species, as a potential target [73],[74]. Studies with *L. sigmodontis* have established that both rifampicin [75] and tetracyclines cause growth retardation and sterilisation of adult worms, but in the latter case daily treatment for at least for 4 weeks is required [76]. In cattle, long-term, intermittent antibiotic chemotherapy with oxytetracycline is macrofilaricidal and worm death is preceded by a considerable reduction in *Wolbachia* [77]. In contrast, short-term, intensive treatment (daily therapy for 2 weeks) induces only transient and inconsequential effects on *Wolbachia* numbers and is not macrofilaricidal [78]. In humans, 6 weeks of treatment with 100 mg per day of doxycycline has resulted in a complete inhibition of embryogenesis from between 18 [74],[79],[80] and 24 months [73]. Logistical considerations and compliance will demand shorter regimes if tetracyclines are to find their way into routine use against onchocerciasis. One approach will be to identify combination therapies. Given that an added benefit of long-lasting sterilisation of female worms will be interruption of transmission, research in this area should be considered a priority. Importantly, the most recent results show that increasing the dose of doxycycline to 200 mg also exhibits a strong macrofilaricidal effect in human onchocerciasis [81],[82]. However, it must also be recognised that there are restrictions on use of this class of antibiotics in young persons and pregnant women. Nevertheless, these observations have intensified strategies to exploit the *Wolbachia* genomes for improved antibiotic targeting [83]. In addition, for final local elimination, e.g., in foci in the Americas, anti-wolbachial chemotherapy is being considered [81].

Targeting *Wolbachia* may also resolve the problem of ivermectin use in areas where onchocerciasis and loiasis are co-endemic and where mass treatment is often discouraged because of severe adverse reactions that result from the rapid destruction of *Loa loa* microfilariae in the central nervous system [84],[85]. *L. loa* does not possess endosymbiont *Wolbachia* [86], and therefore a therapy that targets the bacteria in *O. volvulus* should have no effect on *L. loa*. Targeting *Wolbachia* is arguably the only approach currently available (apart from suramin treatment in a hospital) to treat potentially resistant strains of *O. volvulus.*

## Conclusions

Ten years ago, the mechanisms by which filarial nematodes are killed by the mammalian host were largely unknown. Although fine detail of these processes remain to be determined, the animal models have now allowed us to determine conclusively that Th2 responses drive protective immunity against L3 larvae as well as the microfilarial stage. Bigger weaponry that includes a Th1 pro-inflammatory component may be needed to tackle the adult stage. However, in successful infections all these mechanisms fail because of the ability of the parasite to initiate regulatory pathways. Bypassing this regulation may be the key to development of a vaccine and future disease control. This will require a thorough understanding of how the parasite induces regulation and identification of the targets and processes that mediate a protective but non-pathological response. In the meantime, the prospect of developing new drug regimes using antibiotics to complement ivermectin treatment and to achieve a macrofilaricidal activity may mitigate against problems of emerging drug resistance and offer new therapy in cases where ivermectin is contra-indicated.

**Box 2. Methods**

Selection of the publications cited was based on four approaches:

Direct knowledge of the authors of this manuscript regarding key papers or unpublished research.
Searches online (predominantly PubMed) for filariasis and a relevant keyword. For example, filarial* and regulat* were used to ensure that we were aware of relevant papers when writing about immune regulation.
Searches on line (predominantly PubMed) for information on a particular topic that may not be recalled by a “filaria*” search (for example, “ivermectin resistance”).
Review of the main Web sites that maintain up-to-date information on disease statistics for river blindness and related diseases. These include:
  ○ The WHO: http://www.who.int/topics/onchocerciasis/en/
  ○ The Carter Center: http://www.cartercenter.org/health/river_blindness/index.html
  ○ Sightsavers: http://www.sightsavers.org/What20We20Do/Eye20Conditions/River20Blindness/World1622.html
  ○ Global Partnership to Eliminate Riverblindness: http://www.worldbank.org/afr/gper/

**Box 3. Learning Points**

1. Although ivermectin has made an immense contribution to onchocerciasis control, it cannot abrogate transmission, and its efficacy is threatened by emerging drug resistance. Therefore, a drug that is effective against adult worms, or a vaccine, is required.
2. Progress on understanding protective immunity in onchocerciasis has been accelerated by two model systems in particular, *Litomosoides sigmodontis* in mice and *Onchocerca ochengi* in cattle.
3. In both mice and cattle, immunisation with irradiated third-stage larvae (L3) induces significant protection, providing proof-of-principle for a vaccine. In contrast, drug-abbreviated infections fail to induce protective immunity.
4. *Onchocerca volvulus*, *O. ochengi*, *L. sigmodontis*, and many other filariae contain endosymbiotic bacteria (*Wolbachia*), which, if depleted by prolonged antibiotic chemotherapy, result in adult worm death. Shorter treatment regimens involving drug combinations are being investigated.
5. Immunity against filarial parasites is complex, with Th2-type mechanisms driving protection against L3 and microfilariae, whilst both Th1 and Th2 pathways are involved in resistance to adult worms. Parasite survival is achieved by the induction of an immunoregulatory milieu.
